# A retrospective analysis of time delays in patients presenting with stroke to an academic emergency department

**DOI:** 10.4102/sajr.v22i1.1319

**Published:** 2018-06-21

**Authors:** Diteboho Khalema, Lara N. Goldstein, Susan Lucas

**Affiliations:** 1 Department of Radiology, University of the Witwatersrand, South Africa; 2 Department of Emergency Medicine, Faculty of Health Sciences, University of the Witwatersrand, South Africa

## Abstract

**Background:**

Stroke presents commonly to the emergency department (ED), and is a common cause of morbidity and mortality in South Africa. Early ED presentation and early neuroimaging are required in order for thrombolysis to be a potential therapeutic modality.

**Objectives:**

To determine the time to ED presentation, time to computed tomography (CT) scan and the potential influencing factors for patients with stroke.

**Methods:**

A retrospective record review of all patients who presented with clinical features of stroke to a tertiary academic ED in Johannesburg, South Africa, from 01 January to 31 December 2014.

**Results:**

Data from 232 eligible stroke patients were analysed. The median time to presentation to the ED was 33 h with the majority of patients (81.3%) presenting after the 4.5 h window for thrombolysis. The median time to CT was 8 h. Only 3.9% of patients had a CT scan within one hour of arrival. Patients with loss of consciousness were associated with earlier hospital presentation (*p* = 0.001). None of the patients were thrombolysed.

**Conclusion:**

Patients with stroke commonly present late to hospital. If we are to make a difference in this group of vulnerable patients, further education and training needs to be emphasised regarding ‘time is brain’. Communication and commitment is also required by the emergency medical services, ED and radiology staff in order to prioritise stroke patients and to reduce delays.

## Introduction

Ischaemic stroke is ranked as the third leading cause of death in South Africa.^1^ Guidelines for ischaemic stroke thrombolysis, though controversial, advocate its administration within 3 h of stroke onset (which can be extended up to 4.5 h for certain patient groups).^2,3,4,5^ There are various provisos, however, before this can occur – computed tomography (CT) scan exclusion of an intra-cranial haemorrhage, large ischaemic stroke or other non-stroke diagnoses as well as the exclusion of contraindications to the drug itself.^5^

Despite its availability, there are still low rates of stroke thrombolysis. This is mainly because of time delays in patient presentation to hospital, as well as in-hospital delays such as protracted times in obtaining a CT scan.^6,7,8^ More than 60% of stroke patients present after the recommended 3 h window period in both developing and developed countries.^6,7,8,9,10^

Given the growing burden of stroke worldwide and the paucity of data from the developing world, the aim of this study was to investigate the time from stroke symptom onset to presentation to the emergency department (ED), the time from arrival to CT scan acquisition and the potential influencing factors.

## Methods

This study was a retrospective record review of all patients who presented with clinical features of stroke to a tertiary academic ED in Johannesburg, South Africa, from 01 January to 31 December 2014. The ED has approximately 60 000 patient visits per annum.

### Data collection

**FIGURE 1:**
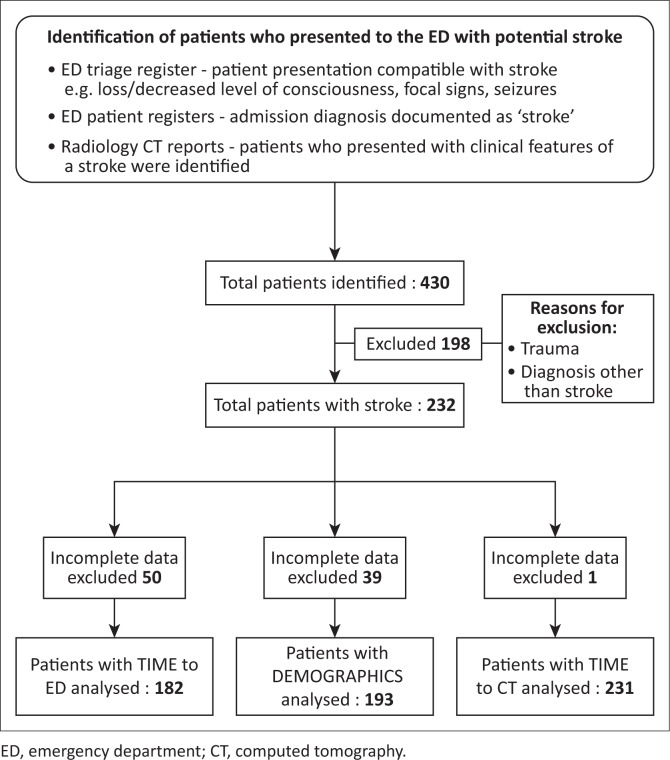
Identification of patients with potential stroke.

Patients presenting with stroke were identified through the ED triage and patient registers, as well as the radiology department CT reports. The files of these patients were then retrieved from the records department and the data captured by a single researcher (D.K.). Trauma patients, patients with a diagnosis other than stroke and patients with incomplete data were excluded (Figure 1).

### Statistical analysis

Categorical variables were presented in percentage format. Continuous variables such as time were presented as medians with interquartile ranges (IQR) (as the time data were positively skewed). The differences in time to ED presentation were evaluated in a number of different subgroups and assessed using the Mann–Whitney test (for two level co-variates) or Kruskal–Wallis test (for more than two levels) for the continuous variables. The subgroups evaluated were sex, smoking, alcohol, medical history and clinical signs.

Data analysis was carried out using SAS (version 9.4 for Windows). The 5% significance level was used throughout.

## Ethical consideration

Ethical approval for the study was obtained from the Human Research Ethics Committee (medical) of the University of the Witwatersrand (M150123).

## Results

Figure 1 shows the inclusion and exclusion of patients available for analysis. The patient demographic data are summarised in Table 1.

**TABLE 1:** Demographics of patients presenting with stroke (*N* = 193).

Characteristics	*N*	%	Mean	SD
Age (years)	-	-	57	15
**Gender**				
Male	86	44.6	-	-
Female	107	55.4	-	-
**Stroke type**				
Ischaemic	145	75.1	-	-
Haemorrhagic	48	24.9	-	-

### Time to emergency department

**FIGURE 2:**
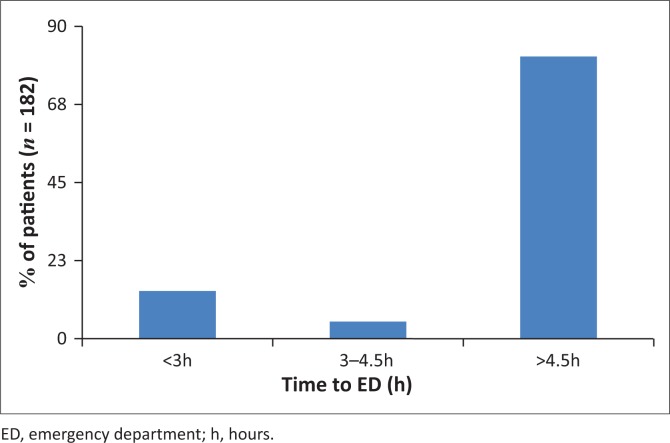
Categorisation of presenting times to emergency department by thrombolytic window periods.

**FIGURE 3:**
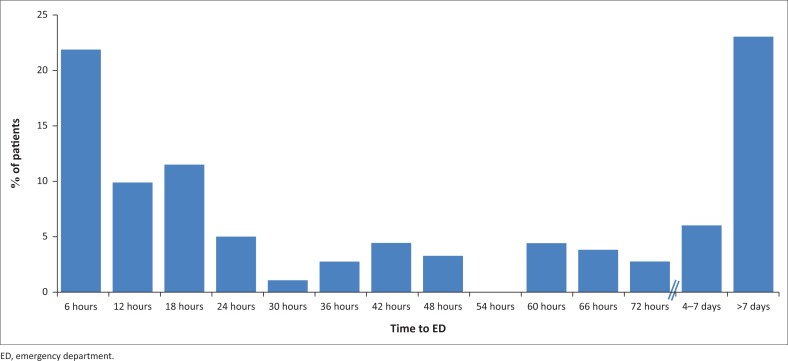
Distribution of presenting times for stroke patients to the emergency department.

The median time to presentation to the ED was 33 h (interquartile range 8 h – 111 h). Categorisation of patient presenting times based on the thrombolytic window period is shown in Figure 2. The actual distribution of patient presentation times in hours is shown in Figure 3.

### Time to computed tomography

**FIGURE 4:**
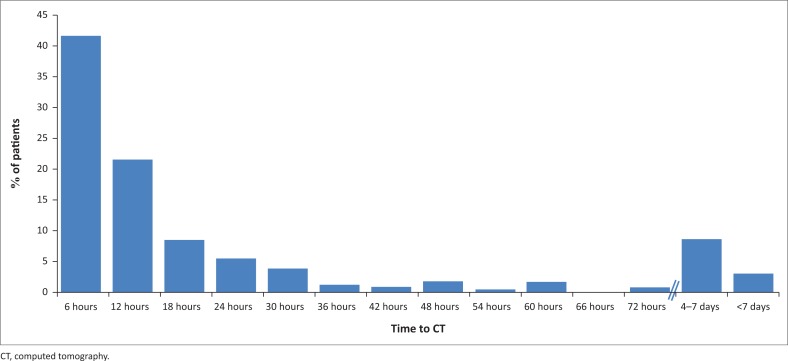
Distribution of presenting times to arrival at computed tomography for stroke patients.

The median time from presentation to the ED to CT was 8 h (interquartile range 4–21 h). There were only nine patients (3.9%) who received a CT scan within one hour of arrival in the ED. Figure 4 demonstrates the distribution of time delays in the patients obtaining their CT.

Tables 2 and 3 show the time delays to ED presentation based on patient demographic characteristics, previous medical history and clinical signs.

**TABLE 2:** Comparison of times to emergency department presentation by subgroup.

Characteristics	Time (h)		*p*
	Median	IQR	
**Gender**			
Male	36.25	10.00–174.20	0.12
Female	17.07	5.50–70.80	
**Smokers**			
No	24.58	6.20–154.10	0.60
Yes	19.83	10.88–58.90	
**Alcohol**			
No	22.75	5.90–85.60	0.33
Yes	46.00	14.58–178.90	

IQR, interquartile range; h, hours.

**TABLE 3:** Comparison of times to emergency department presentation by medical history and clinical signs.

Characteristics	Time (h)				*p*
	Yes		No or unknown		
	Median	IQR	Median	IQR	
**Medical history**						
Hypertension	23.2	7.0–70.8	33.0	6.2–158.0	0.74
Diabetes mellitus	46.3	14.8–189.2	20.0	5.8–84.7	0.05
Hyperlipidaemia	13.2	3.3–69.8	24.6	7.0–111.0	0.43
Previous stroke	16.7	3.4–70.8	24.6	6.6–85.6	0.71
Previous myocardial infarction	4.3	3.4–70.8	23.5	6.9–103.8	0.44
HIV positive	42.6	14.0–103.8	62.9	3.4–70.8	0.30
**Clinical signs**						
Hemiparesis or hemiplegia	33.2	8.0–103.8	18.1	5.6–119.8	0.44
Facial paresis	33.0	8.0–103.8	20.0	5.9–85.6	0.47
Aphasia	23.0	5.8–132.5	31.0	6.6–85.6	0.69
Convulsions	9.8	5.3–47	33.0	7.0–103.8	0.22
Loss of consciousness	6.6	2.7–21.8	36.5	10.0–156.0	0.001*
Elevated blood pressure	19.0	3.0–57.8	38.5	9.8–177.8	0.27
Elevated glucose	71.3	2.7–188.6	23.0	6.7–107.4	0.14

IQR, interquartile range; h, hours.

* , significant value.

## Discussion

The burden of stroke is profound.^11^ Although controversial, the only available potential option for treatment is thrombolysis; however, time constraints and usage precautions make its administration challenging.^2,3,4,5^ Both patient and hospital factors play a role in delays to stroke treatment.

### Demographics

Endogenous oestrogen is commonly deemed to be neuroprotective. The incidence of stroke amongst women appears to increase after menopause as this hormone wanes.^12^ This is reflected in the female preponderance of stroke in our study as well as in other South African stroke studies.^13,14^ In contrast, this is contrary to studies from England, Nigeria, India, China and Australia.^6,7,8,10,15^ There is no clear explanation for this phenomenon.

The median stroke age was similar to the other South African stroke studies.^13,14^ This is younger than in developed countries. This may be related to the increased resources and other fiscal differences associated with developed countries or perhaps because of the higher rate of hypertension in South Africa.^16^

South Africa’s high rate of hypertension may also be a major influencing factor in the higher relative rate of haemorrhagic strokes compared to developed countries.^16^ In the United States, the proportion of all strokes because of ischaemia and haemorrhage are 87% and 13%, respectively.^17,18^

### Time to emergency department presentation

Despite efforts to increase public awareness of stroke, significant delays in seeking care after stroke still occur.^10^ Perhaps there is greater stroke awareness in Gauteng than in the Free State, as Daffue et al. found that only 7.5% of patients presented within the 4.5 h therapeutic window compared to 18.7% of our population. Similar to our findings, the majority of their patients (82%) presented 8 h after symptom onset.^13^

Our presentation time to ED findings almost parallels other low- and middle-income countries like Nigeria^7^ (21%) and China^6^ (25%) but are lower than in developed countries like England^10^ (<3 h presentation rate of 39.5%) and Australia^8^ (31.3% presenting within 4.5 h). Lack of knowledge has been postulated to be a causative factor that has led to various community educational programmes in order to overcome it.^15^ This absence of stroke awareness amongst the population is not the only contributor to this deficit.

Anosognosia is the ‘lack of awareness or the underestimation of a specific deficit in sensory, perceptual, motor, affective or cognitive functioning due to a brain lesion’. Prevalence of anosognosia in stroke varies between 10% and 58% depending on the time since the brain insult.^19^ This cannot account for the vast majority of patients delaying their presentation to hospital, however. It is, perhaps, the psychological defence mechanism of ‘denial’ rather than the pathology-related denial that may lead to these patients avoiding admitting that their symptoms may indeed be a stroke and therefore could be a contributing factor to their delayed presentation.

Logistical difficulties in accessing hospital care could also have led to late patient presentation. Although this is not considered to be a significant contributing factor in developed countries, the difference in access to pre-hospital care in our population is different.^8^ According to Nielsen et al.’s study on the status of pre-hospital care in 13 low- and middle-income countries, including South Africa, large proportions of severely ill and injured people are still not able to receive formal pre-hospital emergency medical care.^20^ In a prospective stroke study in Nigeria that enrolled 81 patients, no patients were brought to hospital by ambulance.^7^ Inability to access an ambulance to transport the patient to the hospital could have caused delays in their presentation. The retrospective nature of our study meant that information regarding this piece of the puzzle was unfortunately not available.

### Time to computed tomography

Early CT is necessary to exclude a haemorrhagic stroke, a large ischaemic stroke or other non-stroke diagnoses, which are all contraindications to thrombolysis.^5^ The amount of time patients spend between the ED and CT can cause significant in-hospital delays, thereby making them ineligible for thrombolysis.^21^

The majority of our patient population had a CT scan performed after 1 h from ED arrival – this is longer than the 25 min recommended by the American Heart Association.^5^ These guidelines have since been amended with a new recommendation which states that ‘brain imaging studies should be performed within 20 min of ED arrival in at least 50% of patients who may be candidates for’ time-sensitive stroke therapies.^22^ In a study conducted by Ogbole et al. in Nigeria, time delays to CT were found to be more significant in patients with an ischaemic stroke than in patients with a haemorrhagic stroke. This was attributed to the more exaggerated symptoms that patients with haemorrhagic strokes display, making them more likely to get their CT sooner.^23^ Maestroni et al. found that patients presenting within 3 h were more likely to have a CT earlier than patients arriving after 3 h.^24^ This was echoed in Canada where patients who presented soon after stroke onset were male, had no history of stroke and arrived at hospital from a setting other than home, had an increased likelihood of timely neuroimaging.^21^ A good working relationship between radiology and the ED, as well as ancillary staff, such as porters and clerks, would be integral to decreasing delays to CT acquisition.

### Patient factors influencing presentation times

Although female patients presented earlier than their male counterparts, this was not statistically significant because of the wide range of presentation times

Smoking and alcohol usage also did not influence presenting times. In contrast to this, medical history did play a role.

### Time to presentation: Medical history and clinical signs

Loss of consciousness was associated with earlier presentation to hospital. A stroke registry review by Jiang et al. in China also found loss of consciousness associated with earlier presentation to hospital – no other associated co-morbidities were found.^25^ Besides the finding of delayed presentation associated with diabetes by Jin^6^, most other analyses of co-morbidities found no association.^7,8,9,10^

Decreased level of consciousness was associated with earlier patient presentation to ED. This is similar to other studies.^6,24^ Unconscious patients may be perceived as having a more serious condition than a patient with unilateral symptoms, rendering the family more likely to bring them in earlier, hence the earlier presenting time. Jin et al. found a higher percentage of patients with haemorrhagic stroke presented with decreased level of consciousness compared to patients with an ischaemic stroke.^6^

Being diabetic excludes patients from the later thrombolysis window.^22^ Therefore, early presentation to hospital and education of stroke symptoms should be particularly emphasised in education to this patient group. That being said, patients known with diabetes have also been shown to have poorer outcomes when thrombolysed for stroke.^26^

The most common presenting symptoms were hemiplegia and facial paresis. These are the most common and easily identifiable stroke symptoms and strong independent predictors of a stroke.^27^ In this study, these factors were not necessarily associated with early presentation in contrast to a study conducted by Gargano et al.^28^ Education may help to address this shortfall.

### Barriers to early stroke presentation and thrombolysis

None of the patients in the study received thrombolysis. A Rwandan study had the same findings.^29^ The biggest challenge was that patients arrived at hospital after the thrombolysis window period and were therefore not eligible. Twenty-five (13.7%) of our patients could have potentially received thrombolysis having presented within 3 h and a further nine patients in the 4.5 h time window – if they had met the inclusion criteria.

As this was a retrospective study, patient knowledge was not assessed. Williams et al. found that 75% of stroke patients could not accurately identify their symptoms as a stroke. Patients who had a stroke previously were more likely to identify their symptoms as a stroke but did not necessarily present early to hospital. Even patients with risk factors for stroke were not aware of symptoms and the available treatment of stroke.^30^ At-risk patients and their families should be educated about compliance to medication, changing of lifestyle and early presentation, should they develop a stroke.

Anosognosia, denial and logistical issues are patient-based factors that could have contributed to delayed presentation. In-hospital delays can potentially be decreased with good communication and a team approach to management.

## Limitations of the study

Patient files and CT reports that could not be recovered limited the sample size for time to ED presentation and patient demographics. Patient times (time of stroke onset, time of ED arrival and time to CT) were not always documented. These limitations were mainly as a result of the retrospective nature of the study.

## Conclusion

In a South African academic hospital setting, the vast majority of stroke patients had a delayed presentation to hospital outside the window for thrombolysis.

Public education to improve awareness of early symptoms of stroke and the available treatment including the time limitations could improve the management of stroke. Communication and commitment is also required by the emergency medical services, ED and radiology staff in order to prioritise stroke patients to reduce delays.
